# Flexible, stretchable, and single-molecule-sensitive SERS-active sensor for wearable biosensing applications†

**DOI:** 10.1039/d3ra03050d

**Published:** 2023-07-11

**Authors:** Muhammad Aminul Haque Chowdhury, Nishat Tasnim, Mainul Hossain, Ahsan Habib

**Affiliations:** a Department of Electrical and Electronic Engineering, University of Dhaka Dhaka-1000 Bangladesh mahabib@du.ac.bd

## Abstract

The development of wearable sensors for remote patient monitoring and personalized medicine has led to a revolution in biomedical technology. Plasmonic metasurfaces that enhance Raman scattering signals have recently gained attention as wearable sensors. However, finding a flexible, sensitive, and easy-to-fabricate metasurface has been a challenge for decades. In this paper, a novel wearable device, the flexible, stretchable, and single-molecule-sensetive SERS-active sensor, is proposed. This device offers an unprecedented SERS enhancement factor in the order of 10^11^, along with other long-desired characteristics for SERS applications such as a high scattering to absorption ratio (∼2.5) and a large hotspot volume (40 nm × 40 nm × 5 nm). To achieve flexibility, we use polydimethylsiloxane (PDMS) as the substrate, which is stable, transparent, and biologically compatible. Our numerical calculations show that the proposed sensor offers reliable SERS performance even under bending (up to 100° angles) or stretching (up to 50% stretch). The easy-to-fabricate and flexible nature of our sensor offers a promising avenue for developing highly sensitive wearable sensors for a range of applications, particularly in the field of personalized medicine and remote patient monitoring.

## Abstract 2

Our novel wearable sensor offers unprecedented sensitivity (enhancement factor ∼10^11^) for remote patient monitoring. It holds great potential for advancing personalized medicine and improving patient care.

## Graphical abstract

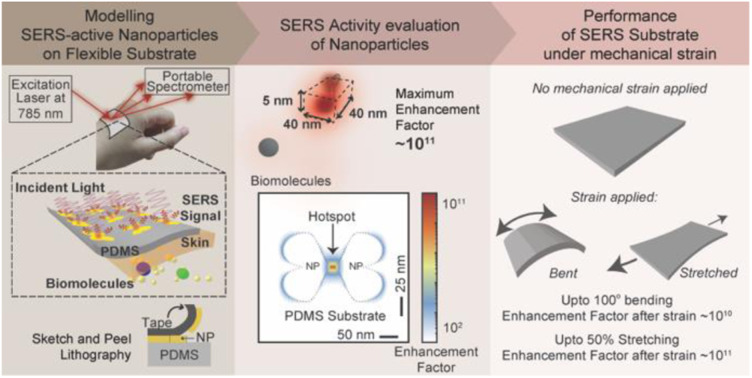

## 1 Introduction

When it comes to the non-invasive, real-time monitoring of an individual's biometrics, wearable sensors are a revolutionary development in diagnostic technology.^1^ Starting with the first demonstration of wearable sensing in 1962, when the Holter Monitor measured the electrical activity of the heart, wearables have achieved several milestones and may now compete with the performance of sophisticated medical instruments.^1,2^ Over the years, pioneering works have lead to wearable biosensors that are capable of elucidating the dynamics of the human body at the molecular level. Recently, flexible wearable sweat sensors have attracted attention due to the potentials of use in point-of-care, remote patient monitoring applications and personalized medicine.^3,4^ Sweat is rich in various electrolytes, metabolites, hormones and other biomolecules that can serve as a biomarker in various diagnostic applications.^5^ Accurate real-time monitoring of the level of these analytes can reveal significant information about the physiological condition of a patient. Moreover, wearable sensors for drug monitoring has attracted special interest in personalized medicine.^6^ Conventionally, patients are prescribed drugs in standardized doses, regardless of their individual drug metabolism profile and other clinical conditions. Customizing the dose of a drug according to the patient's metabolic profile can greatly enhance the efficiency of therapy while reducing its side effects.^7,8^ A number of different categories of sweat-based biosensors have been explored over the years showing considerable advances in this field.^9^ For example, wearable electrochemical sensors are a convenient and portable alternative to sizeable and complex laboratory setups. However, electrochemical sensors are slow, unstable and often only detect one type of analyte at a time, posing a considerable challenge in developing a real-time, multiplexed, and label-free sensing platform with universal target specificity.^10–13^

Several groups have recently demonstrated the promise of Surface-Enhanced Raman Scattering (SERS) in next-generation wearable sensors due to its very sensitive, multiplexed chemical detection of complex analytes. SERS enables non-invasive structural fingerprinting of extremely low-concentration analytes through the use of Localized Surface Plasmons (LSP)-mediated amplification of electrical fields or chemical enhancements.^14,15^ Wang *et al.*^16^ demonstrated a flexible wearable SERS sensors consisting of a metasurface made with Ag nanocube on hydrogel substrate that can monitor extremely low concentration of various drugs (lidocaine, cocaine and methotrexate) from sweat in real time. Liu *et al.*^17^ reported a gold nanomesh wearable SERS sensor that can measure concentration of various analytes like uric acid, MDMA, Triazolam *etc.* Koh *et al.*^18^ fabricated a flexible SERS sensor consisting of silver nanowire metasurface and demonstrated real time monitoring of 2-fluoro-methamphetamine. Santos *et al.*^19^ reported fast detection of paracetamol using SERS by utilizing gold nanoparticles. Apart from label free detection of biomolecules from sweat, SERS paves the way for rapid and *in vivo* diagnosis of a plethora of important biomarkers with specificity by functionalization of the nanoparticles as utilized in SERS Tags or MIP-SERS sensors.^20,21^ Sun *et al.*^22^ demonstrated the noninvasive detection of microRNA-21-5p in cerebrospinal fluids. This biomarker has shown significance in the prognosis and monitoring of disease progression in patients with subarachnoid hemorrhage. The detection was achieved by utilizing a gold nanostar SERS probe. Berus *et al.*^23^ reported the detection of various bacterial species (*N. gonorrhoeae*, *U. urealyticum etc.*) causing sexually transmitted disease using SERS-based sensors. Moreover, SERS detection strategy has been utilized by Arabi *et al.*^24^ for enantiomeric discrimination, which is crucial in biosensing, pharmacological and toxicity analysis.^25^ However, despite the promising applications of SERS in wearable sensors, the development of a highly sensitive flexible SERS-active plasmonic metasurface that is comfortable for long-term usage and conforms to the skin has remained a significant challenge. This objective has been extensively pursued for years, aiming to address various crucial aspects and considerations. These include achieving high near-field enhancement, creating a large hotspot volume with an optimal nanogap between two metal nanoparticles, maintaining a high scattering-to-absorption ratio, utilizing a highly flexible and breathable substrate, and ensuring reliable SERS activity of the metasurface under different deformations.

Here, we introduce the flexible, stretchable, and single-molecule-sensitive SERS-active (F3S) sensor, based on a plasmonic heart-shaped metasurface, that offers several desirable characteristics for wearable SERS sensors. The metasurface provides a strongly enhanced electric field, a large plasmonic hotspot-filled volume, a high scattering-to-absorption ratio, a flexible and breathable substrate, and reliable SERS signal measurement under various deformations. To achieve high electric field enhancment, we employ the strong capacitive coupling of surface plasmons oscillating in heart-shaped plasmonic nanodimers with nanometer-sized gaps that suppress the decay of plasmon resonance. Our numerical calculations demonstrate that this plasmonic metasurface provides a maximum SERS enhancement factor in the order of 10^10^ to 10^11^, making it suitable for single-molecule-level detection. The heart-shaped structure's unique plasmonic response provides a large scattering efficiency, leading to a significantly improved scattering-to-absorption ratio (∼2.5). Moreover, our device features a flexible and breathable substrate made of polydimethylsiloxane (PDMS). The combination of the optimized heart-shaped plasmonic structure and PDMS substrate ensures high mechanical robustness under various deformations. Our calculations reveal that the maximum enhancement factor remains relatively constant at 10^10^ even for bending angles up to 100° compared to the unbent structure. Furthermore, our metasurface maintains a nearly unchanged maximum enhancement factor even when stretched to 50% of its original size. Therefore, our flexible, stretchable, and single-molecule-sensitive SERS-active metasurface offers remarkable properties for next-generation wearable sensors. Additionally, recent advancements in machine/deep learning-assisted Raman spectroscopy have the potential to enhance the applications of our proposed wearable sensors, enabling the analysis and detection of different molecules from multiplexed Raman spectra.^26^ With its unprecedented sensitivity and ability to meet desirable characteristics, our metasurface holds great promise in revolutionizing the field of wearable SERS sensors, contributing to real-time remote patient monitoring and personalized medicine.

## 2 Device structure & methodologies

**Fig. 1:**
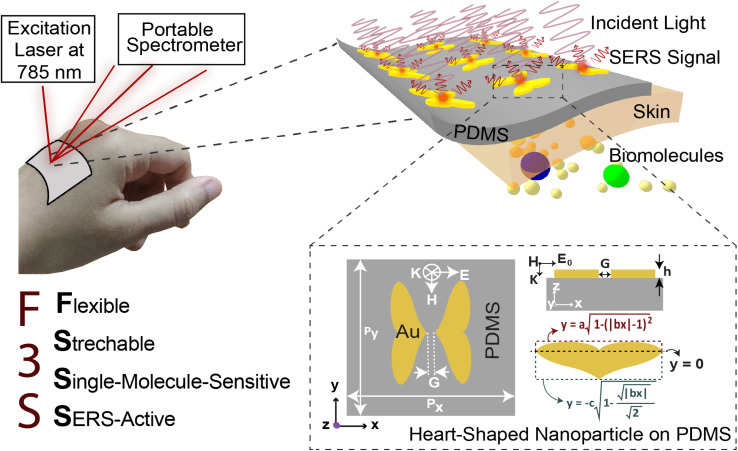
Concept illustration of the proposed plasmonic SERS sensor that can be laminated on the wrist and used with a portable Raman spectrometer for label-free biochemical (sweat) analysis in real-time. The top inset shows the heart-shaped nanodimer array on a flexible PDMS substrate for sweat analysis. The bottom inset shows the modeling of the heart-shaped NP. *G* is the nanogap between the dimer, P*x*, and P*y* periodicity along the *x* and *y* direction, respectively, and *h* is the height of the NP along the *z*-direction.

The near-field enhancement factor is a crucial parameter for achieving high SERS efficiencies. Our sensor structure is inspired by a recently reported gold nanodimer of heart-shaped nanoparticles (NPs) with sub-10 nm gap sizes inversely designed by using a topology optimization algorithm.^27^ The topology optimization algorithm shows that a gold nanodimer of heart-shaped nanoparticles exhibits a substantially high electric field enhancement compared to other shapes like nanorods, nanodisks *etc.* In this study, we propose fabricating heart-shaped gold nanoparticle dimers on a PDMS substrate to create wearable SERS sensors with high flexibility, stretchability, and biointegratability (Fig. 1). The experimental set up of our proposed wearable sensor is straightforward consisting of a laser source and a portable Raman spectrometer. By illuminating the laser on the flexible metasurface attached on human skin (preferably sweaty by stimulating the sweat glands^4^), the enhanced Raman signal from sweat can be readily captured using the spectrometer. Furthermore, we optimized the NP dimer's dimensions to obtain superior SERS capabilities. The heart shape can be modeled using the analytical equations with three parameters *a*, *b*, and *c*, as follows:1
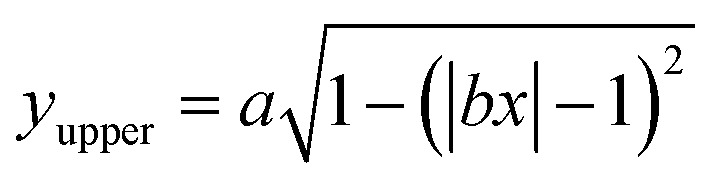
2
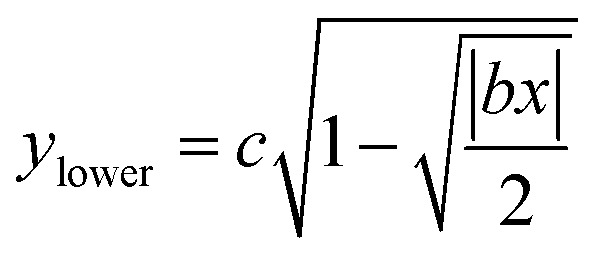


Eqn (1) and (2) were used to design the upper half and the lower half of the heart shape respectively (Fig. 1, inset). The three parameters *a*, *b*, and *c* in the analytical equations used to model the heart shape have specific geometrical meanings (Fig. S1, and ESI Text 1†). The parameter *a* represents the distance between the cusp of the heart and the center of the circle that generates the cardioid curve. The parameter *b* represents the distance between the center of the circle and the curve itself, which determines the curvature of the heart. Finally, the parameter *c* represents the distance between the center of the circle and the midpoint of the line segment connecting the cusp to the bottom of the heart. The aspect ratio (AR), which is determined by the following equation, was used to investigate how the plasmonic response depends on the shape of the heart NP:3
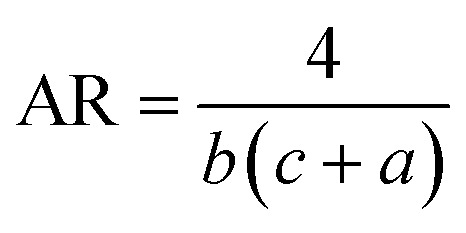


**Fig. 2:**
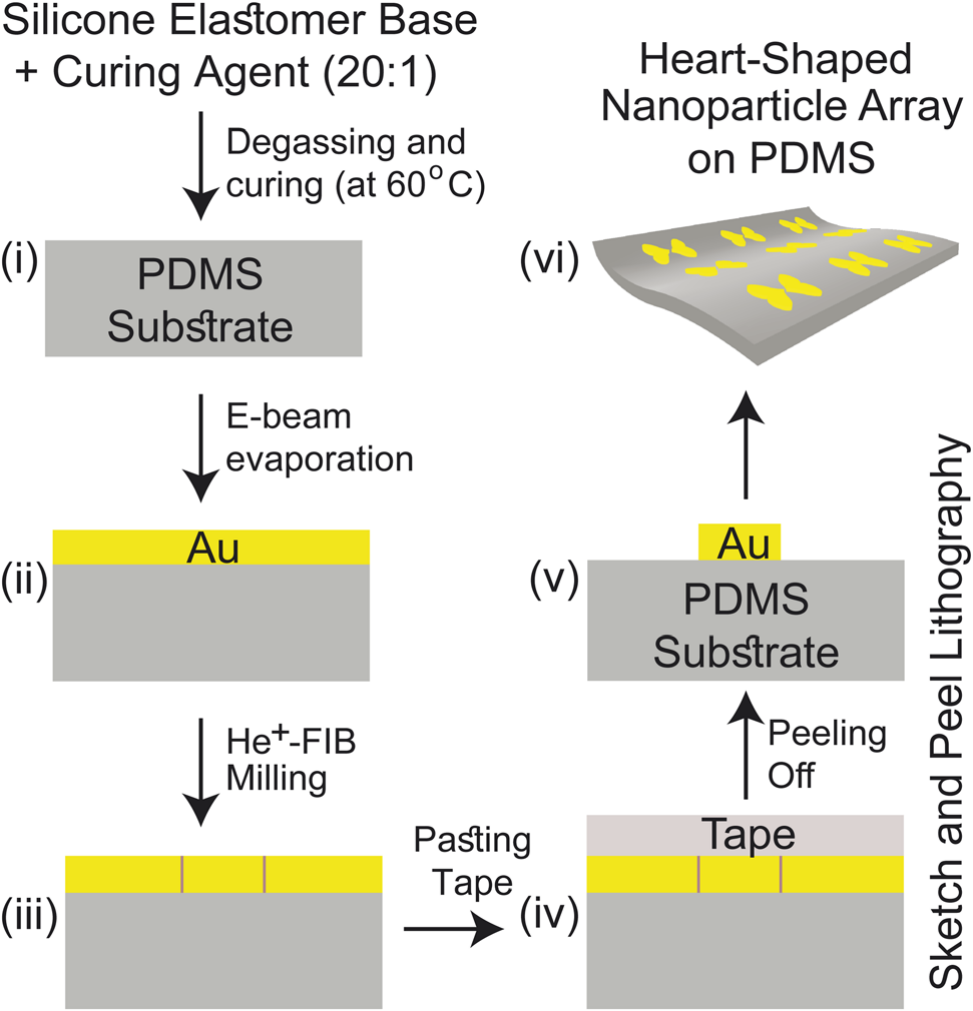
The proposed fabrication process for the wearable sensor is depicted in a scheme utilizing the “sketch and peel” lithography. The process includes (i) schematics of the PDMS substrate, (ii) deposition of a 45 nm thick Au layer on the substrate *via* e-beam evaporation, (iii) transfer of a heart-shaped pattern using He^+^-FIB milling, (iv) application of sticky tape, and (v) selective peeling of the outside film to leave the internal target structure on the PDMS substrate. (vi) Three-dimensional view of the sensor.

Our proposed fabrication route for the metasurface utilizes the state-of-the-art focused helium ion beam milling (He^+^-FIB) technique and the recently reported “sketch and peel” lithography to achieve nanoparticle dimers with an ultra-small gap size of 5 nm. This fabrication method, which has been extensively described in the literature,^27–29^ was to create the metasurface comprising the cardiac dimer structure. A detailed discussion of the fabrication process is provided in ESI Text 2,† and a summary is presented in Fig. 2. The PDMS substrate can have their self-healing, elasticity, modulus, and transparency properties modified based on their composition before polymerization.^30,31^ Gold nanoparticles (Au-NP) on PDMS substrate is robust and stable while offering reliable flexibility and the storage time of the metasurface can extend from more than 15 days up to two months if kept at 4 °C, preferably in an N_2_ ambient.^32–34^ Furthermore, surface modifications on PDMS can enhance the adhesion of active materials such as gold (Au) that can lead to improved stability of the proposed metasurface.^31,35^

The scattering cross-section *σ*_scat_ at a specific wavelength was calculated by integrating the radially outward Poynting vector *S*_scat_ across an auxiliary surface containing the nanoparticle dimers and then dividing by the incident irradiance *I*_inc_(*λ*) as follows:^36,37^4
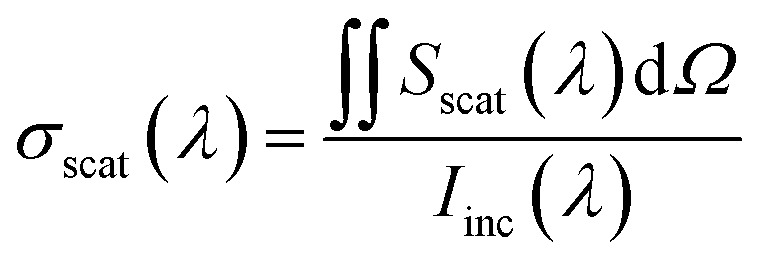


Similarly, the extinction cross-section *σ*_ext_ was calculated as follows:5
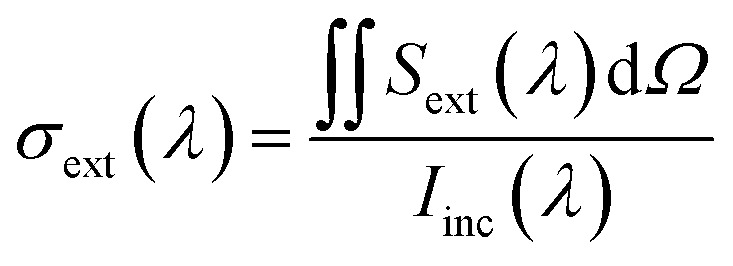


The absorption cross-section *σ*_abs_ can be calculated from *σ*_scat_ and *σ*_ext_ as follows:

6 *σ*_abs_(*λ*) = *σ*_ext_(*λ*) − *σ*_scat_(*λ*)

The absorption and scattering efficiencies^38^*Q*_scat_ and *Q*_abs_ are the cross-section of absorption and scattering normalized by per unit area, *A* = *a*π/*b* + 32*c*/15*b* (Fig. S1 and ESI Text 1†):7
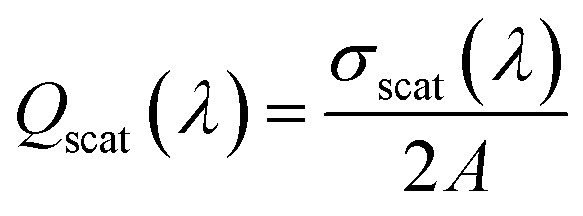
8
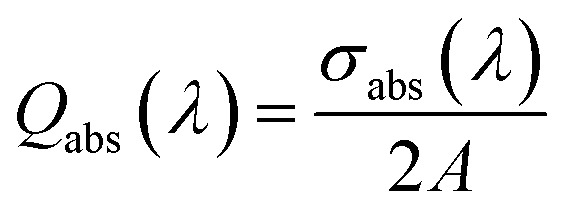


SERS signal enhancement can be classified into two categories: electromagnetic and chemical, with the former being more significant.^39^ The electromagnetic enhancement is attributed to the substantial increase in the electric field (*E*) at the molecule's position compared to the incident electric field (*E*_0_). This enhancement can be quantified using the highest SERS enhancement factor (EF_max_), which is calculated as calculated as follows:^40^

9 EF_max_(*λ*) ∼ |*E*(*λ*)/*E*_0_(*λ*)|^4^

To better estimate the SERS performance of the suggested structure, we calculated the average local enhancement factor (EF_avg_) over a volume *V* as follows:^41^10
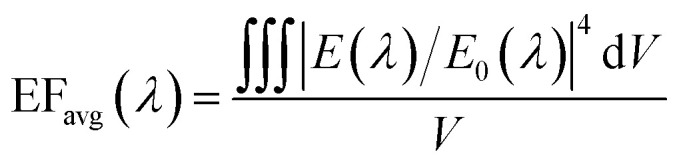


**Fig. 3:**
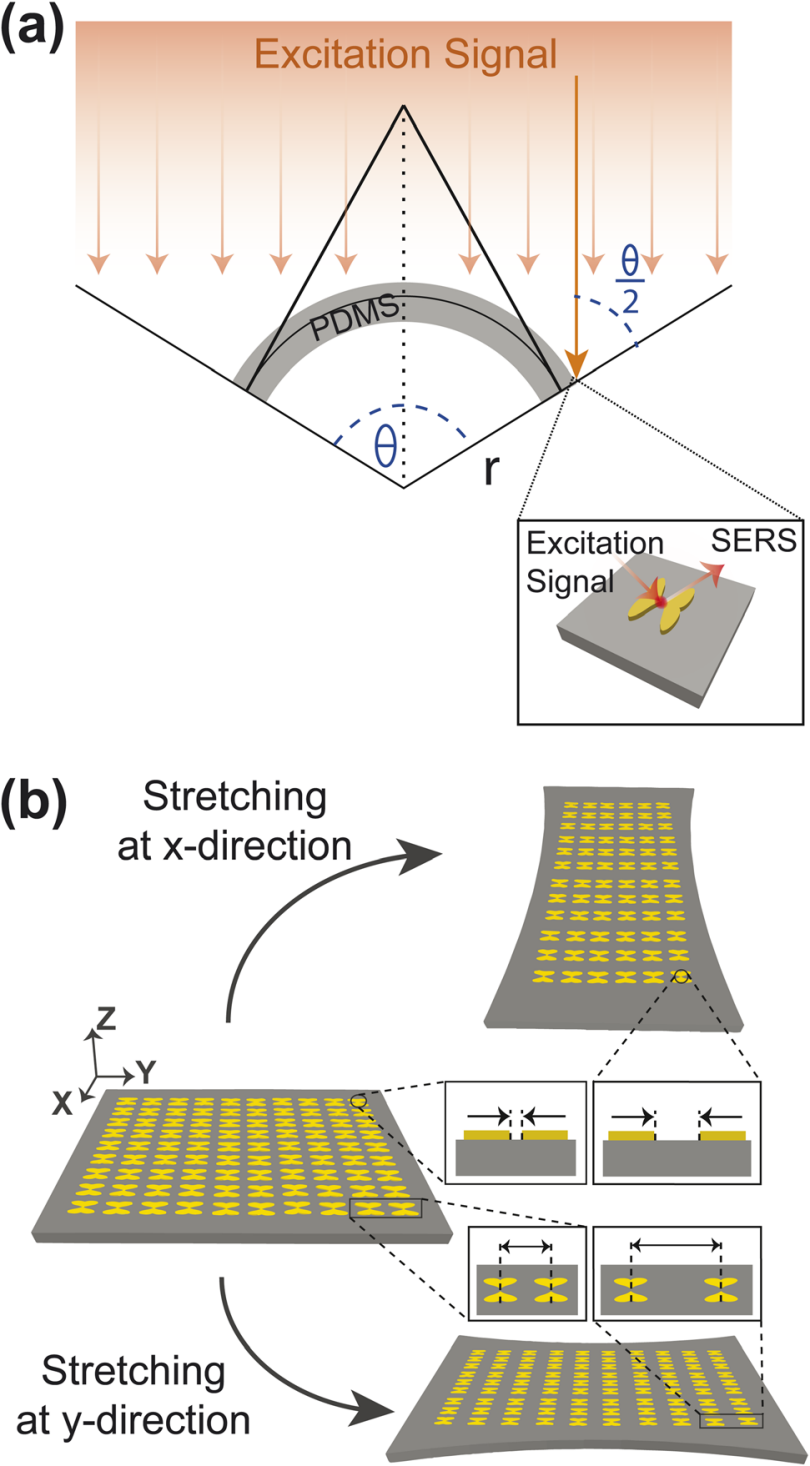
Modelling of (a) bending (b) stretching to approximate the SERS performance of the proposed metasurface.

To ensure flexibility, stretchability, and biocompatibility, our proposed metasurface is made up of PDMS. As shown in Fig. 3(a), we bend the surface with a radius of curvature of *r* and *a* bending angle of *θ* to simulate the effect of bending. The bending of the metasurface causes the excitation laser beam to be incident on the NP dimers obliquely. The NP dimers at the edge of the metasurface, at an angle of *θ*/2, are least exposed to the incident light. Therefore, we measure the SERS improvement by shining light at an angle of *θ*/2 onto the NP dimers. On the other hand, Fig. 3(b) shows that when the substrate is stretched in either in the *x* or the *y* direction, the periodicity also changes accordingly. Assuming that the substrate is stretched evenly everywhere, stretching in the *x* direction makes the nanogap bigger.

We used the Ansys Lumerical FDTD solutions to run the Finite-Difference Time-Domain (FDTD) simulations.^42^ For gold, the real and imaginary parts of the refractive index (*n*, *k*) from the work of Johnson and Christy were used.^43^ The *n*, *k* values of PDMS (20 : 1) were used as reported by Zhang *et al.*^44^ The change in the refractive index of PDMS under tension and bending is negligible.^45^ As a result, we neglected the refractive change caused by bending and stretching in our simulations. The background index was set to 1. The conformal variant 1 (CMT-1) was chosen for mesh refinement because the simulation involves metals.^46^ On the *x* and *y* boundaries, we used periodic boundary conditions, and on the *z* boundaries, we used steep angle PML boundary conditions with 12 layers. When the light came from an angle, Bloch boundary conditions were used in the *x* and *y* directions, and standard PML boundary conditions with 24 layers were used in the *z*-direction. In our proposed structure, the plane wave source is polarized along the *x*-axis and propagated along the negative *z*-axis to hit the metasurface from the front. From one side of the source area, the plane wave sources hit the *xy* plane NP dimers with constant electromagnetic energy. Around the metal NPs, a 2.5 nm × 2.5 nm × 2.5 nm mesh was used, and around the nanogap, a 1 nm × 1 nm × 1 nm mesh was used. Electric field and power monitors in the frequency domain were used to record the simulation results.

## 3 Results & discussion

**Fig. 4:**
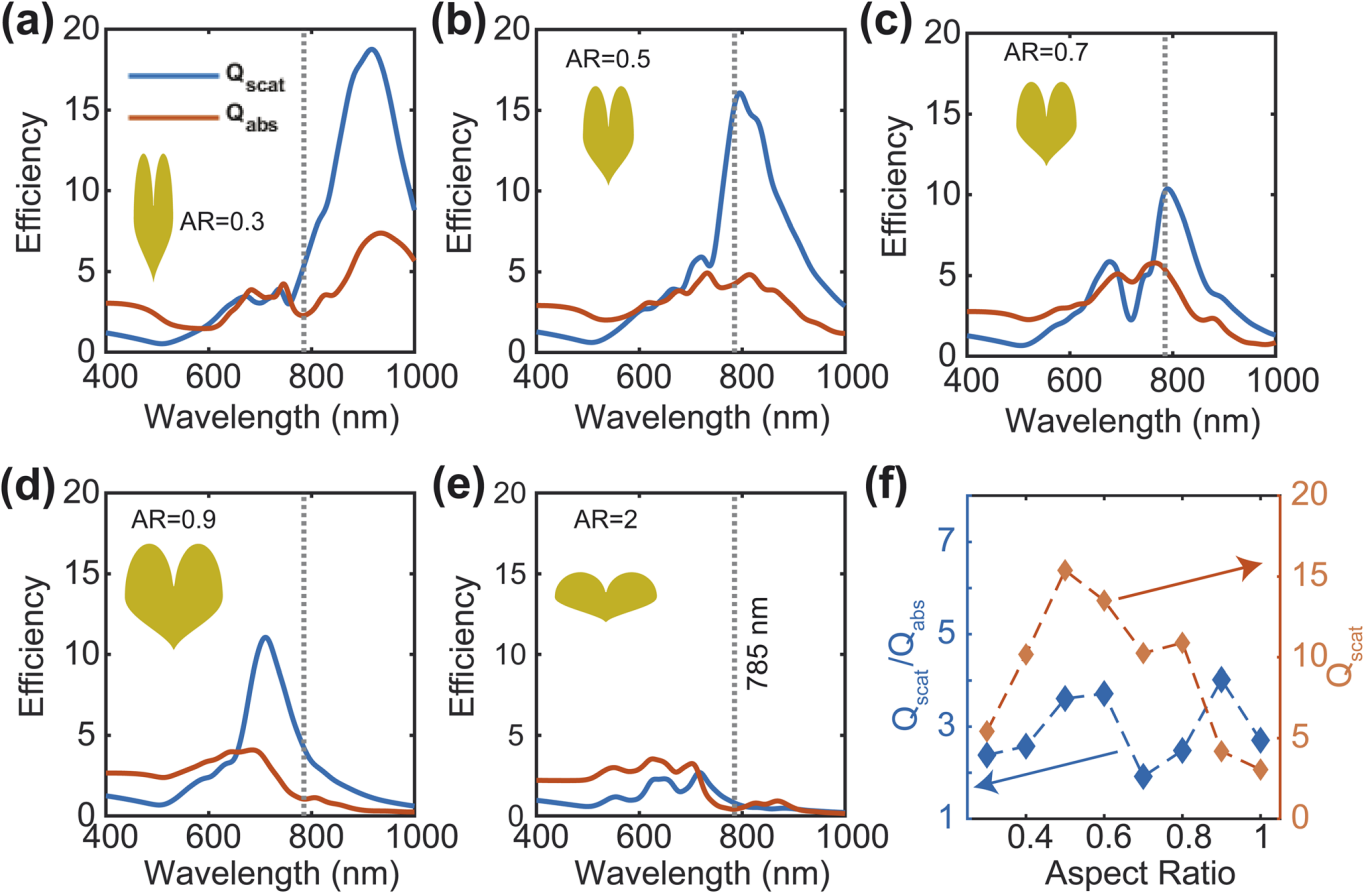
Scattering and absorption efficiency for different AR (a–e). Scattering to absorption ratio and Scattering efficiency for different AR at 785 nm wavelength (f). The height of the NP is 45 nm.

The efficiency and effectiveness of the F3S device rely heavily on the scattering-to-absorption ratio and local field enhancement of the plasmonic nanostructure. A high scattering-to-absorption ratio, which is the ratio of scattering efficiency to absorption efficiency, and a high local field enhancement are desired to achieve a high signal-to-noise ratio (SNR) of the Raman signal.^47^ To study the scattering-to-absorption ratio of the F3S device, we calculate the scattering efficiency *Q*_scat_ and absorption efficiency *Q*_abs_ using eqn (7) and (8), respectively. Initially, we vary the aspect ratio (AR) of the heart-shaped NP dimer to evaluate its plasmonic efficiency while keeping the xyz-plane area constant (Fig. 4(a)–(e)). Our calculations indicate that the heart-shaped dimer scatters more efficiently when the AR is small (Fig. 4(a)–(d)). For higher AR values, the scattering and absorption efficiencies become almost identical (Fig. 4(e)). Furthermore, our calculations reveal that the peak wavelength shifts towards the blue with increasing AR (Fig. 4(a)–(e) and ESI Fig. S2†). The plasmonic peak resonance shifts towards the blue with increasing aspect ratio due to changes in the local electric field distribution and the interaction between the incident electromagnetic field and the plasmonic nanostructure. As the aspect ratio increases, the geometry of the nanostructure changes, altering the distribution of the local electric field and causing the shift. The relationship is non-linear and influenced by various factors such as size, shape, and material properties.^48^

**Fig. 5:**
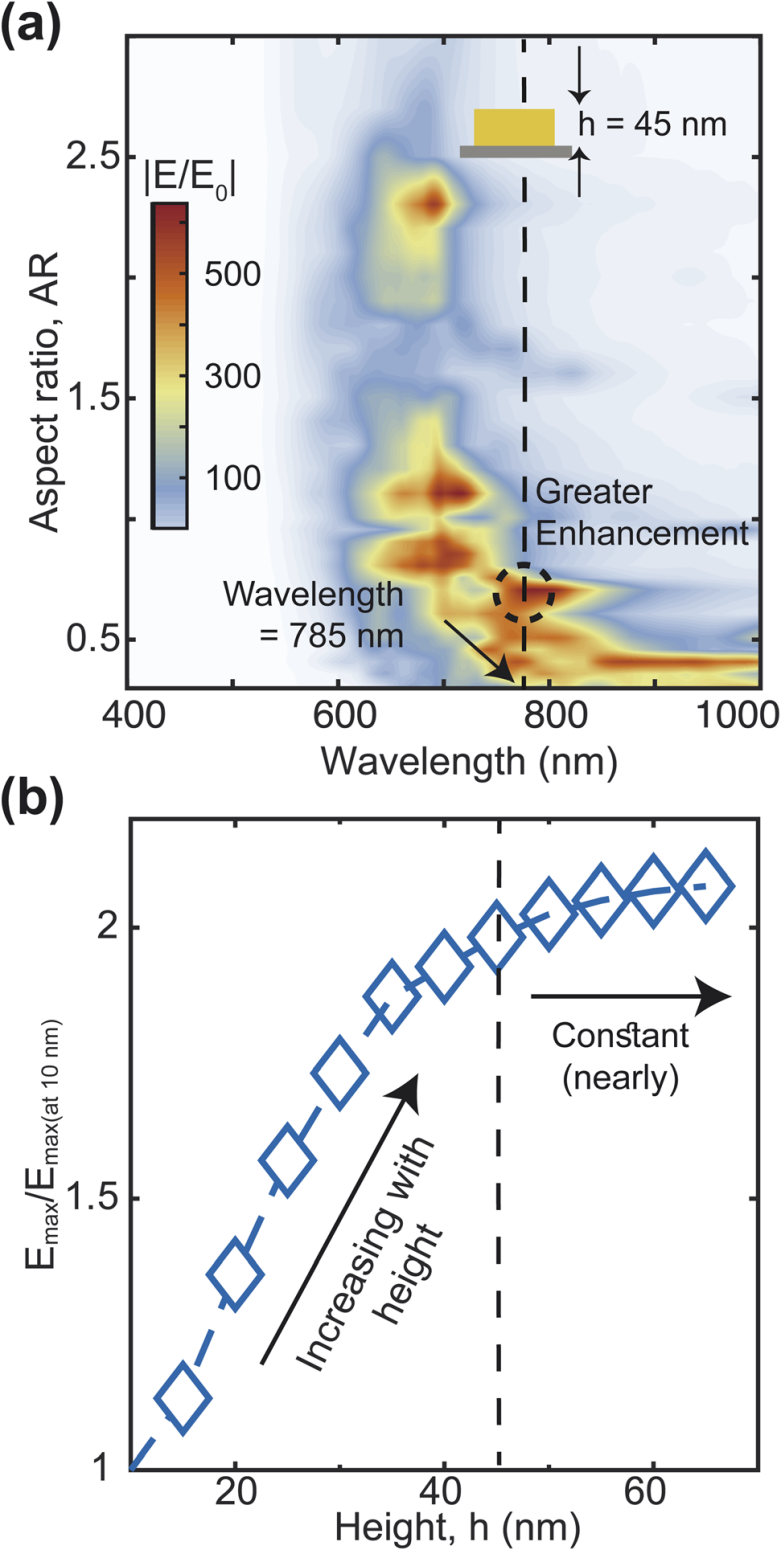
Electric field enhancement map for various aspect ratios (a). Field enhancement at 785 nm wavelength and 0.7 aspect ratio for different heights (b).

Next, we calculate the scattering-to-absorption ratio and scattering efficiency for different AR values at the commonly used Raman spectroscopy wavelength of 785 nm (Fig. 4(f)). Based on our results, when the AR values fall within the range of 0.4 to 0.8, the scattering efficiency is more than twice that of the absorption efficiency. To further optimize the AR of the heart-shape, we analyze the enhancement of the local electric field with respect to the aspect ratio. We calculate the electric field enhancement at different wavelengths for various aspect ratios of the heart-shaped NPs (Fig. 5(a), and ESI Fig. S3†). Our results show that the high electric field enhancement (>300) is achieved when the aspect ratio is less than 1.5 (Fig. 5(a)). Additionally, we find that heart-shaped NPs with an aspect ratio of 0.75 or less provided highest electric field enhancement (>500) in the vicinity of our target wavelength of 785 nm. More specifically, we observe that at a wavelength of 785 nm, the electric field is amplified more than 600 times for an aspect ratio of about 0.7. Based on these findings, we conclude that the most suitable range of aspect ratio for our heart-shaped nanoparticles lies between 0.5 and 0.75.

We have also examined the impact of the height of the plasmonic nanostructure on electric field enhancement, while keeping the aspect ratio fixed at 0.7 and the excitation wavelength at 785 nm (Fig. 5(b)).The maximum electric field enhancement increases rapidly at first and then levels off as the height of the NP increases. The curve flattens out at a height of 45 nm, and at this point, the electric field enhancement is about twice as strong as when the height is 10 nm. Therefore, based on these findings, we decide to use a NP height of 45 nm for further analysis.

**Fig. 6:**
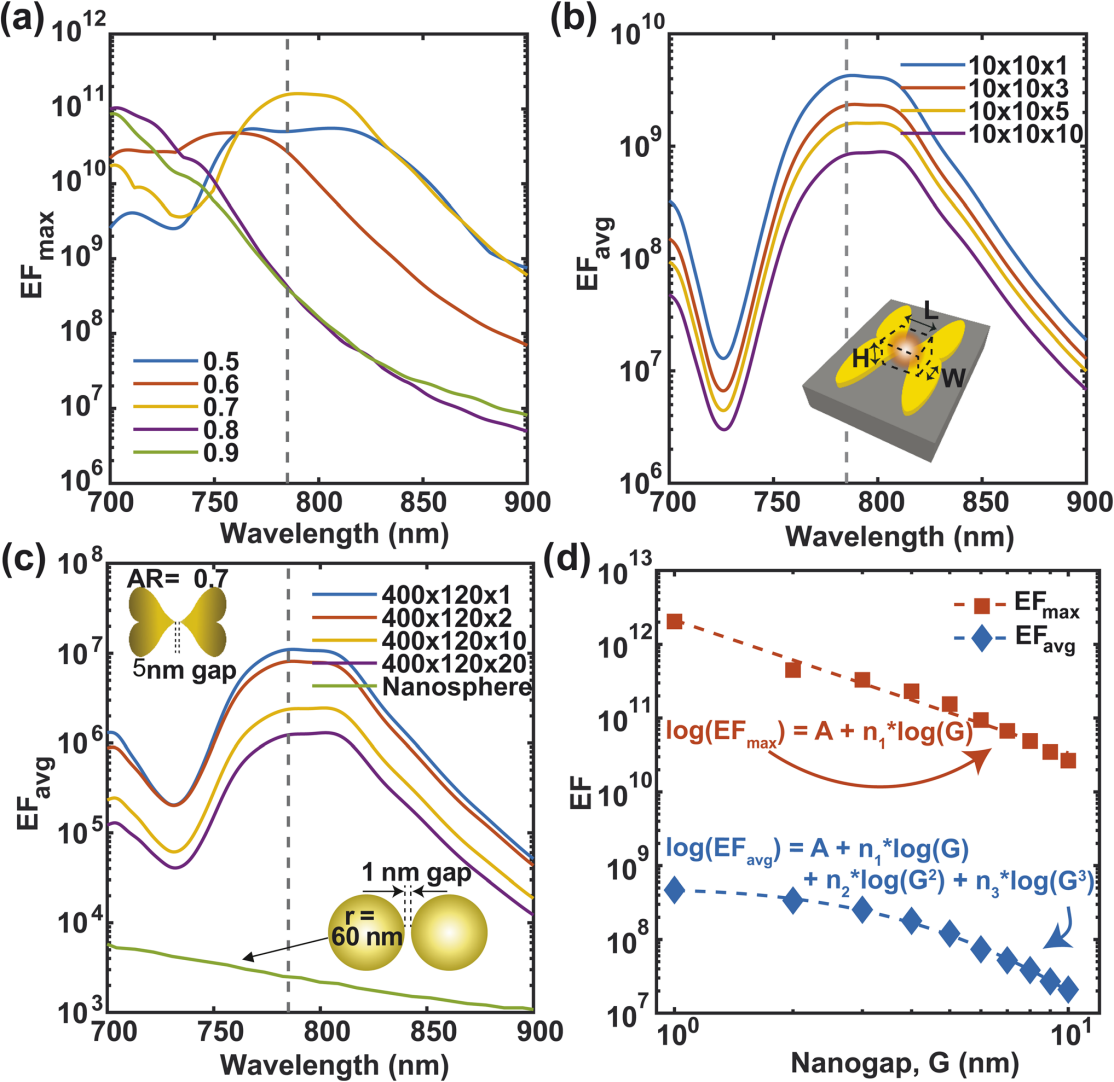
Maximum EF at different wavelengths for different aspect ratios (a). Average EF over different volumes for 0.7 aspect ratio at a wavelength of 785 nm (b and c). Log–log plots of EF_max_ and EF_avg_ as a function of nanogap, *G* at 785 nm wavelength (d). The fitting parameters are listed in Table 2.

Our optimized F3S device could enable a large SERS enhancement factor. To show this, we calculate the maximum SERS enhancement factor EF_max_ (eqn (9)) for heart-shaped dimers with different aspect ratios from 700 nm to 900 nm wavelength (Fig. 6(a)). At 785 nm wavelength (shown by the vertical line), the maximum EF is in the order of 10^11^ for the three aspect ratios (0.5, 0.6 and 0.7) which is usually sufficient for single molecule level detection of various resonant and non-resonant biomolecules.^49,50^ The values of parameters *a*, *b*, and *c* for these three aspect ratios as defined in eqn (1) and (2) are given in Table 1. As we can see, an AR of 0.7 offers slightly better performance in terms of the enhancement factor, so we decided to use it for the remaining analysis in this work. The averaged enhancement factor EF_avg_, as determined by eqn (10), is shown over various volumes close to the nanogap in Fig. 6(b). A hotspot volume of approximately 10 × 10 × 10 nm^3^ results in an average enhancement factor of up to 10^9^. The EF_avg_, which considers the entire area of the NP dimer that molecules come into contact with when they approach the NP from inside the PDMS substrate, is shown in Fig. 6(c). A volume with the dimension 400 nm × 120 nm × 1 nm gives an average enhancement factor of the order of 10^7^, which is quite high considering the very large volume. The EF_avg_ lowers by only one order when the volume is increased 20 times (400 nm × 120 nm × 20 nm = 960 000 nm^3^). In contrast, for gold nanosphere dimers with 1 nm gap and a radius of 60 nm, considering a volume within 2 nm of the metal surface (a volume of around 940 000 nm^3^) gives an enhancement factor^41^ three orders of magnitude lower than the proposed structure. In addition to the comparison with gold nanosphere dimers, we have also evaluated the SERS performance of rod-like, triangular, and elliptical shaped nanoparticle dimers, keeping the volume and aspect ratio same (ESI Text 5†). The maximum enhancement factor (EF_max_) and the average enhancement factor (EF_avg_; over a volume of 10 nm × 10 nm × 10 nm) for these structures are presented in ESI Fig. S4.† The results clearly demonstrate that our proposed heart-shaped nanoparticle exhibits orders of magnitude higher enhancement factors, especially in the vicinity of the 785 nm wavelength, compared to the other structures.

**Table 1:** Parameters *a*, *b*, and *c* associated with different aspect ratios (AR) of the heart shape

AR	*a*	*b*	*c*
0.5	77.0798	0.0513	78.8656
0.6	69.9288	0.0468	72.5213
0.7	65.7702	0.0434	65.8953

Furthermore, we would like to highlight that our proposed heart-shaped NP dimer exhibits a similar level of enhancement on other substrates, such as polymethyl methacrylate (PMMA), polyethylene terephthalate (PET), and polyimides (PI) (ESI Text 6† for details). The comparison of SERS enhancement factors on PDMS, PMMA, PET, and PI substrates is presented in Fig. S5.† This comprehensive analysis demonstrates the versatility and effectiveness of our proposed structure across different substrate materials, further supporting its potential for various wearable biosensing applications.

**Fig. 7:**
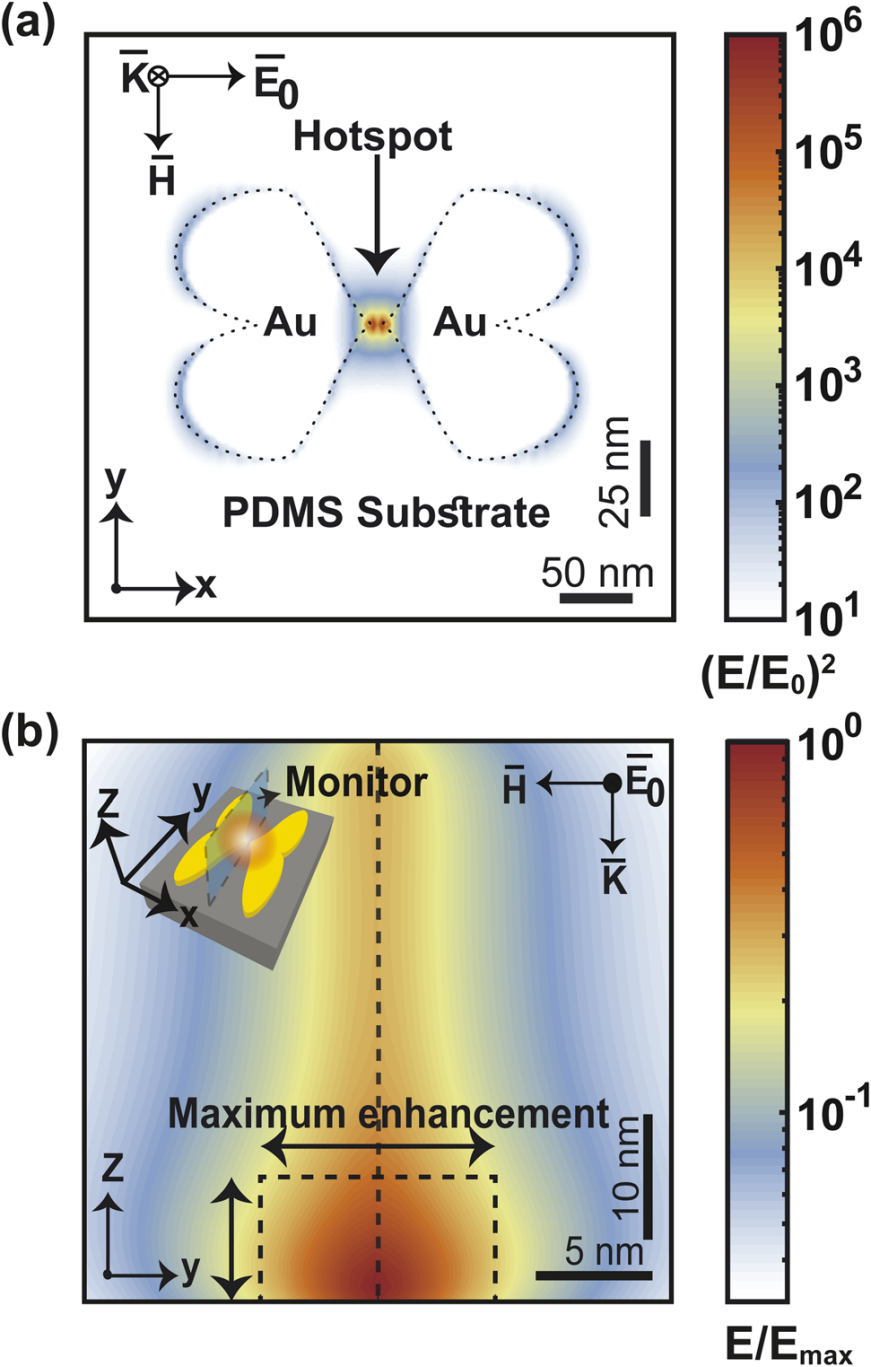
Profile of electric field intensity at *z* = 0 (a). Change in maximum EF as vertical distance from the substrate increases (b).

The sensitivity of SERS devices depends on the nanogap size, with smaller gaps producing higher enhancement factors.^51^ However, the fabrication of smaller gaps requires more advanced and expensive techniques, which poses a challenge for device optimization. We optimize the nanogap of our F3S device by calculating the maximum and average enhancement factors for varied nanogap sizes within a volume of 10 nm × 10 nm × 10 nm (Fig. 6(d)). Our findings indicate that the maximum enhancement factor exhibits a nearly linear relationship in a log–log plot. The near-field enhancement of the heart-shaped NP dimer exhibits a weak but general power-law dependence on the nanogap size. This dependence can be expressed as EF_max_ = 10^A^·*G*^*n*_1_^, where the slope of log(EF_max_) against log(*G*) is approximately −1.801. Remarkably, the slope value we obtained is less steep than the values reported for other nanostructure dimers, such as −2.8, −4, and −4.46,^50,52–54^ indicating that the heart-shaped NP dimer is less susceptible to changes in nanogap size. This is advantageous because achieving an extremely small nanogap can be challenging due to fabrication constraints. Furthermore, we have observed that the average enhancement does not follow a linear trend in the log–log plot. Specifically, as the nanogap size decreases below 2 nm, the EF_avg_ becomes almost flat. This behavior can be attributed to the formation of a hotspot due to charge accumulation at the tip of the heart-shaped structure, as revealed by the electric field intensity distribution in Fig. 7(a). When the nanogap is very small, the surface plasmon is confined to a small volume, leading to an extremely high maximum enhancement factor.^55^ However, the spread of the hotspot shown in Fig. 7(b) decreases as the nanogap size decreases, which results in a deviation of the average enhancement from the maximum enhancement trend. To fit the EF_avg_ data in the log–log plot, we have employed the equation log(EF_avg_) = *A* + *n*_1_ log(*G*) + *n*_2_ log(*G*)^2^ + *n*_3_ log(*G*)^3^, with the fitting parameters presented in Table 2.

**Table 2:** Fitting parameters for equations EF_max_ = 10^A^·*G*^*n*_1_^ and log(EF_avg_) = *A* + *n*_1_ log(*G*) + *n*_2_ log(*G*)^2^ + *n*_3_ log(*G*)^3^, showing the trend of Surface Enhanced Raman Scattering (SERS) enhancement factor as a function of the nanogap, *G*

SERS enhancement factor	*A*	*n*_1_	*n*_2_	*n*_3_	*R* ^2^
EF_max_	12.33	−1.801	—	—	0.9777
EF_avg_	9.51	−0.1142	−0.6727	−0.5798	0.9981

Our F3S device stands out when compared to other cutting-edge wearable SERS sensors. In Table 3, we present a comparison between our proposed metasurface and previously reported wearable SERS sensors, based on their respective SERS enhancement factors. Our metasurface, with a relatively larger nanogap size of ∼5 nm, demonstrates a SERS enhancement factor that is two to three orders of magnitude higher than that of Ag nanocube dimers with a 1 nm gap, as reported by Wang *et al.*^16^ Similarly, our proposed metasurface shows significant improvement when compared to the Ag nanovoids array reported by Zhu *et al.*^56^ and Liu *et al.*^17^

**Table 3:** SERS enhancement factors and plasmonic nanostructures for different references

Reference	SERS EF	Plasmonic nanostructure
Wang *et al.*, *Sci. Adv.*, 2021 (ref. 16)	∼10^7^	Ag nanocube
Zhu *et al.*, *Small*, 2022 (ref. 56)	∼10^8^	Ag nanovoid array
Liu *et al.*, *Adv. Opt. Mater.*, 2022 (ref. 17)	∼10^8^	Au nanomesh array
This work	∼10^10^–∼10^11^	Au heart-shaped dimer

**Fig. 8:**
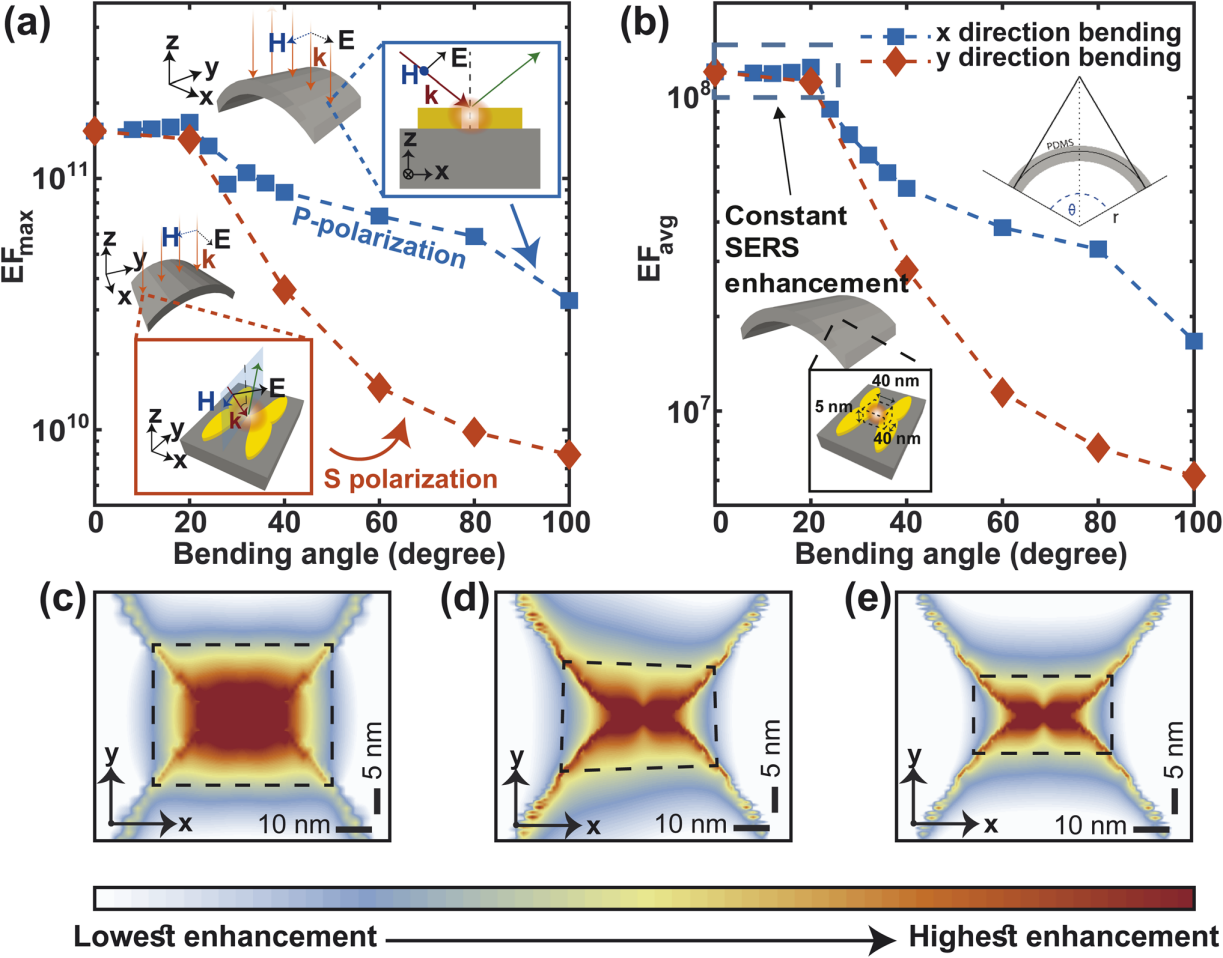
Changes in SERS enhancement factor, EF_max_ (a) and EF_avg_ (b) due to bending. The electric field intensity at the nanogap for three different case, unbent metasurface (c), 100° bending in the *x*-direction (d) and 100° bending in the *y*-direction (e).

Mechanical robustness under various deformations (like bending, stretching, *etc.*) is crucial for skin-based wearable sensors. Reasonable SERS activity should continue to be reliable and stable to guarantee the viability and effectiveness of the wearable sensor. We have approximated the effect of bending the metasurface on the SERS performance, considering the oblique incidence of light on the bent structure (Fig. 8). Our calculation shows that both the maximum (EF_max_) and average (EF_avg_) SERS enhancement factors remain constant up to 20° angle of bending in the *x*- and *y*-direction (Fig. 8(a) and (b)). The SERS EF is more susceptible to bending in the *y* direction than in the *x* direction. The reason for this difference in SERS performance between bending in the *x* and *y* directions is related to the polarization of the incident light and how it interacts with the heart-shaped nanoparticles (NPs). In bending in the *x* direction, the electric field of the incident light is P-polarized (parallel to the plane of incidence; Fig. 8(a), top inset). In contrast, the electric field of incident light is S-polarized while bending in the *y*-direction (perpendicular to the plane of incidence; Fig. 8(a)-bottom inset). P-polarized light causes stronger plasmonic coupling at the nanogap between heart-shaped NPs because the incident light is already polarized in the direction of the plasmonic response. In contrast, when the incident light is S-polarized, the electric field of the incident light is not aligned with the resonant oscillations of the electrons in the NPs. This misalignment can lead to weaker plasmonic coupling and a lower Raman enhancement factor. Nonetheless, for bending angles of 100° angle, the maximum enhancement factor remains in the order of 10^10^, which is typically adequate for single molecule level detection^49^ (Fig. 8(a)). The average enhancement factor only decreases by one order of magnitude in comparison to the unbent structure (Fig. 8(b)). Additionally, for both *x* and *y* direction bending of 100° angles, the electric field distribution at the nanogap remains nearly symmetrical(Fig. 8(c)–(e)). This implies that, even in the bent structure, high enhancement is quite uniform in the vicinity of the apexes of both the heart shaped NP, and thus, more molecules close to the hotspot can give enhanced Raman signals.^57^

**Fig. 9:**
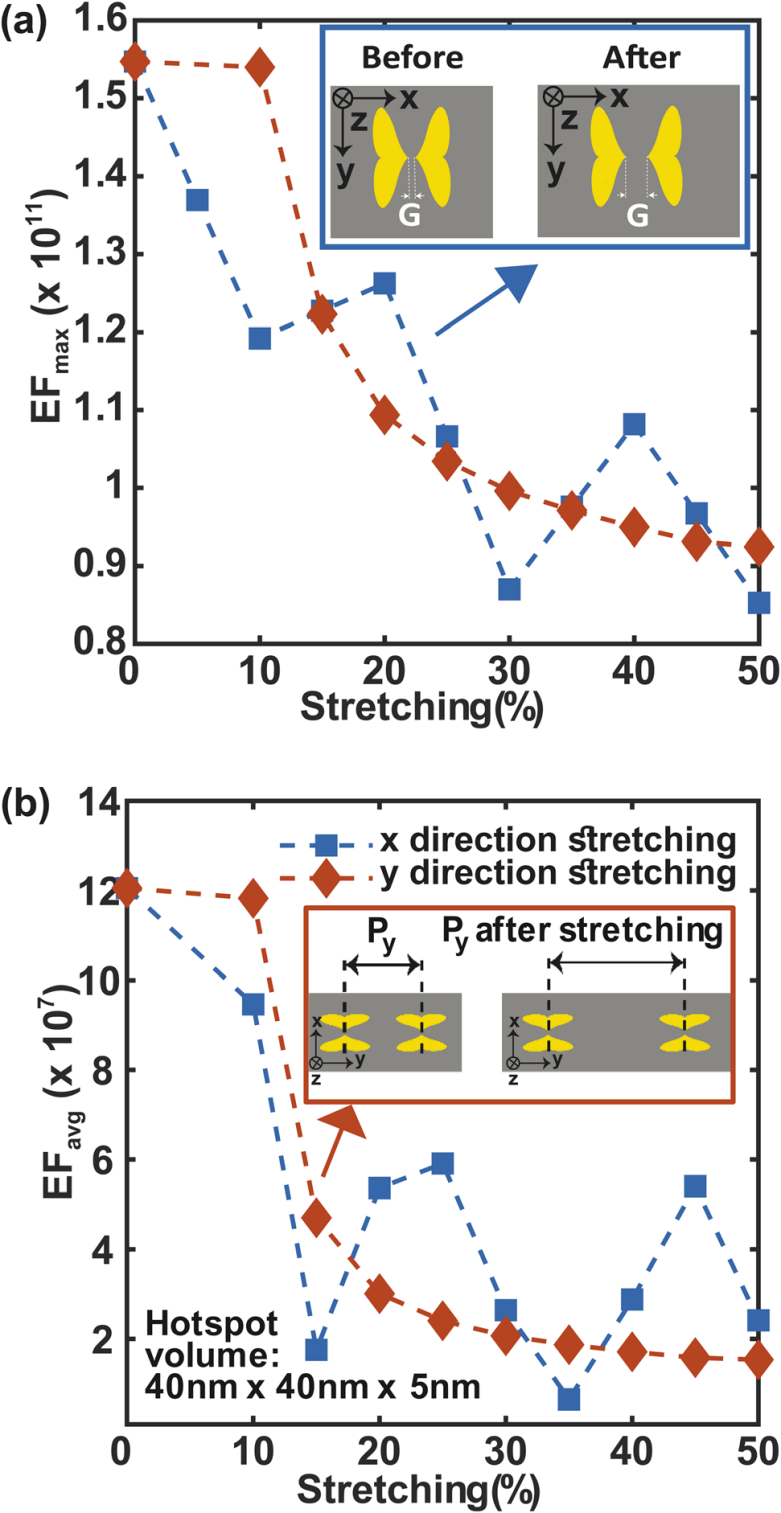
Changes in SERS enhancement factor, EF_max_ (a) and EF_avg_(b) due to stretching.

Stretching is also an essential component of flexibility. We study the effect of stretching on F3S device by accounting for the change in periodicity and near- and far field behavior of the metasurface along the *x* and *y*-directions (Fig. 9). For up to 10% stretching in *y*-direction, maximum and average enhancement factors remain constant and then follow a similar decreasing trend (Fig. 9(a) and (b)). This trend can be explained by considering the near and far fields behaviors of the metasurface. The near field effects from the apex of the heart NP at the nanogap drops more quickly than the far-field. Thus, the plasmonic coupling between the neighboring NP dimers along the *y*-direction is due to the far field contribution.^58^ In contrast to the *y*-direction case, stretching the metasurface in the *x*-direction reduces the maximum SERS enhancement factor more quickly (Fig. 9(a)). Stretching in this direction increases the nanogap as well as the periodicity (Fig. 9(a)-top inset) which results in this steeper drop. The average enhancement factor decays in a similar manner (Fig. 9(b)). Nonetheless, even for a large 50% stretch, the maximum enhancement factor in both cases is only 1.5 times less than that of the unstretched metasurface (Fig. 9(a)), and the average enhancement factor drops by only one order of magnitude (Fig. 9(b)) compared to the unstretched metasurface. It is worth noting that by using a guard ring with the metasurface,^16^ the effect of stretching may be significantly compensated. This makes the structure more appealing for SERS sensing applications that require flexibility and *in vivo* detection.

## 4 Conclusion

In conclusion, we have shown in this work that our proposed F3S device, consisting of a heart-shaped NP dimer array with a favorable aspect ratio on a PDMS substrate, can set a new standard in the pursuit of the ideal flexible SERS-active metasurface by possessing some of the highly sought-after and long-awaited characteristics. Our FDTD simulation indicates that it has a maximum SERS enhancement factor in the order of 10^11^ while maintaining a maximum scattering-to-absorption ratio of more than 2.5 at 785 nm wavelength that promises a high signal-to-noise ratio of Raman signal, promising single molecule level detection for the majority of the biomolecules. Furthermore, it has a large hotspot volume, and the average SERS enhancement outperforms conventional structures by orders of magnitude. The F3S device also exhibits promising SERS performance under a variety of deformation conditions such as bending and stretching, which is critical for wearable device feasibility. Unprecedented sensitivity and reliability can be achieved by using our F3S sensor as a SERS active sensing platform in wearable sensors, paving the way for realizing convenient point-of-care diagnostics, real-time monitoring and precision medicine.

## Author contributions

The research was designed by A. Habib. The theoretical understanding and simulations were developed by M. A. H. Chowdhury, N. Tasnim, M. Hossain, and A. Habib. The main parts of the manuscript were written by M. A. H. Chowdhury, N. Tasnim, and A. Habib. All authors suggested ways to improve the theoretical validation and discussed the results.

## Conflicts of interest

There are no conflicts to declare.

## Supplementary Material

RA-013-D3RA03050D-s001
