# Supplementary material for: Flexible, stretchable, and single-molecule-sensitive SERS-active sensor for wearable biosensing applications
Source: RSC Adv. 2023 Jul 11;13(30):20787–98. doi: 10.1039/d3ra03050d (PMC10334262; doi:10.1039/d3ra03050d)
Supplement: RA-013-D3RA03050D-s001 [file RA-013-D3RA03050D-s001.pdf]

Electronic Supplementary Material (ESI) for RSC Advances.
This journal is © The Royal Society of Chemistry 2023

Journal Name

ARTICLE TYPE

Cite this: DOI: 00.0000/xxxxxxxxxx

F3S Sensor: A Single-Molecule Sensitive Metasurface for Wearable Biosensing Applications

Muhammad Aminul Haque Chowdhury,[†a] Nishat Tasnim,[†a] Mainul Hossain,[a] and Ahsan Habib *[a]

Supplementary Text 1: Structural Parameters of the heart shape

The structure of the dimer has been designed using three key structural parameters: a, b and c. Fig. 1 shows the geometrical significance of the parameters. These structural parameters give the width and length of each nanoparticle, which later helps to calculate the aspect ratio.

Using these parameters, the area of the shape can be calculated. The following equations give the total area, A of the heart shape that has been used, where A_1 is the area of the upper half of the shape, and A_2 is for the lower half of the shape:

$$A_1 = 2\int_0^{2/b} a\sqrt{1-(|bx|-1)^2}\,dx = \frac{a\pi}{b} \tag{1}$$

$$A_2 = 2\int_0^{2/b} c\sqrt{1-\sqrt{\frac{|bx|}{2}}}\,dx = \frac{32c}{15b} \tag{2}$$

$$A = A_1 + A_2 = \frac{a\pi}{b} + \frac{32c}{15b} \tag{3}$$

According to equation S1 and equation S2, the Area A_1 is the part of the entire shape in the positive Y-axis region. Likewise, the area A_2 is the part of the shape in the negative Y-axis region. As the shape extends from -$\frac{2}{b}$ to $\frac{2}{b}$ along the X-axis, the width, $W = \frac{4}{b}$. And the shape extends from -c to a, along Y-axis, making the length, $L = c + a$. These values together, give the aspect ratio of the hear shape as given by equation(3) of the main text.

For a particular aspect ratio, r, and a particular area of the shape, A the value of the parameters a, b and c can be derived by solving the following equations simultaneously:

$$a = \frac{(15Arb2)-128}{rb(15\pi-32)} \tag{4}$$

$$b = \frac{4}{r(c+a)} \tag{5}$$

$$c = \frac{-15(-4\pi + Arb^2)}{rb(15\pi-32)} \tag{6}$$

[a] *Department of Electrical and Electronic Engineering, University of Dhaka, 1000, Dhaka, Bangladesh; E-mail: mahabib@du.ac.bd*
†These authors contributed equally to this work.

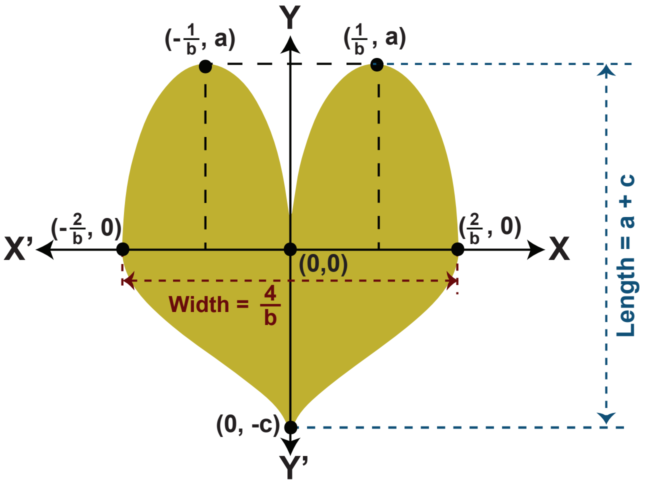

Fig. 1 A graphical outline of the heart shape, marking the structural parameters a, b and c, along with the width and length of the nanoparticle.

In our simulations, we have kept the area of the heart shape as 8000 nm^2.

Supplementary Text 2: Preparation of heart-shaped Gold nanopartcile (Au-NP) on PDMS

To fabricate the heart shaped Au-NP dimer with 5 nm gap, we propose the "Focused Ion Beam" (FIB) milling based "Sketch & Peal Lithography" (SPL) technique developed by Chen *et. al.*[1]. This enables, as demonstrated by this group, rapid patterning of arbitrary shaped plasmonic nanostructures with high-fidelity that are often difficult to achieve with conventional FIB milling process. The fabrication process is as follows:

(a) Determining the outline of the cardiac structure with specific aspect ratio: We have modeled the cardiac shape with analytical mathematical equation as given in the equation (1) and (2) of the manuscript and discussed in details in Supplementary Text-1. The corresponding parameters with specific aspect ratios, studied in this paper are also given in Table S1 of the supplementary. Once, a specific aspect ratio is selected, equation (1) and (2) can be used to determine the (x, y) coordinates of the outline of the cardiac shape

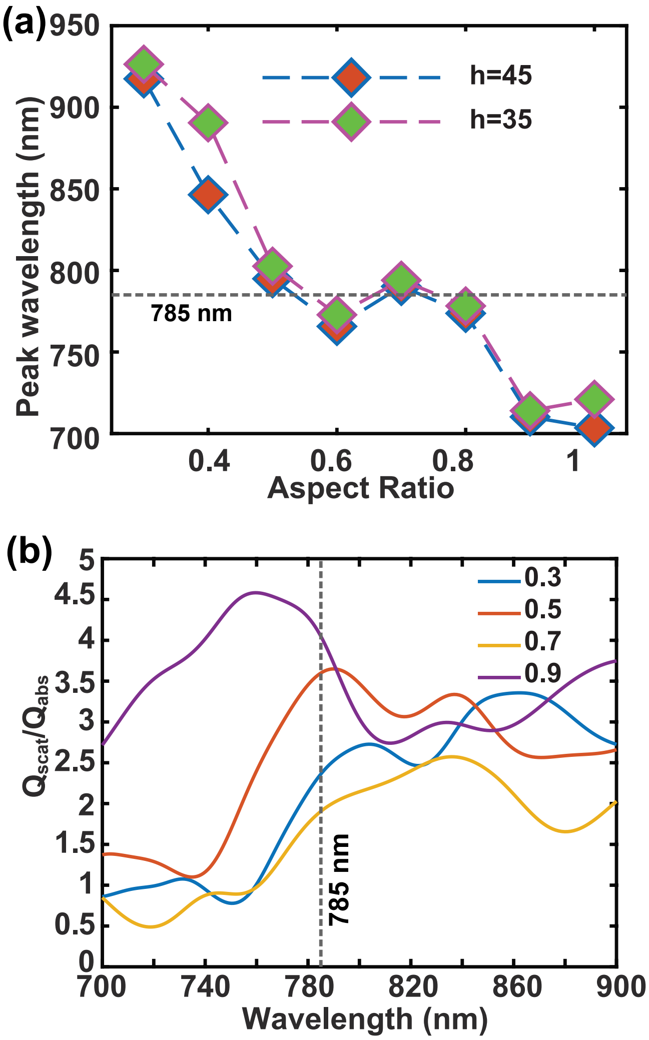

Fig. 2 Dependence of Plasmonic characteristics on aspect ratio of the heart-shaped NP- Peak wavelength of Scattering efficiency for two different heights (a), Scattering to Absorption ratio at various wavelengths for different aspect ratios(b).

structure for milling process.

(b) Preparation of PDMS substrate: PDMS is fabricated by a mixture of main agent and a curing agent. The ratio of these agents (main: curing) determines the optical and mechanical properties of the PDMS. The preparation and effect of this ratio on PDMS layer is discussed by Zhang. *et. al.*[2]. In our proposed wearable sensor, we have used the PDMS layer with 20:1 ratio.

(c) Deposition of Gold (Au) layer on PDMS: Gold layer with specific thickness is to be then deposited in the PDMS substrate. This can be achieved by using electron-beam evaporator system (i.e Lab-Line, Kurt J. Lesker Company etc.)

(d) Focused He+ Ion Beam Milling: The determined outline in step-a now can be realized in the deposited Au layer by He+-FIB using a digital pattern generator, which will isolate the heart-structured nanoparticle from the continuous Au layer

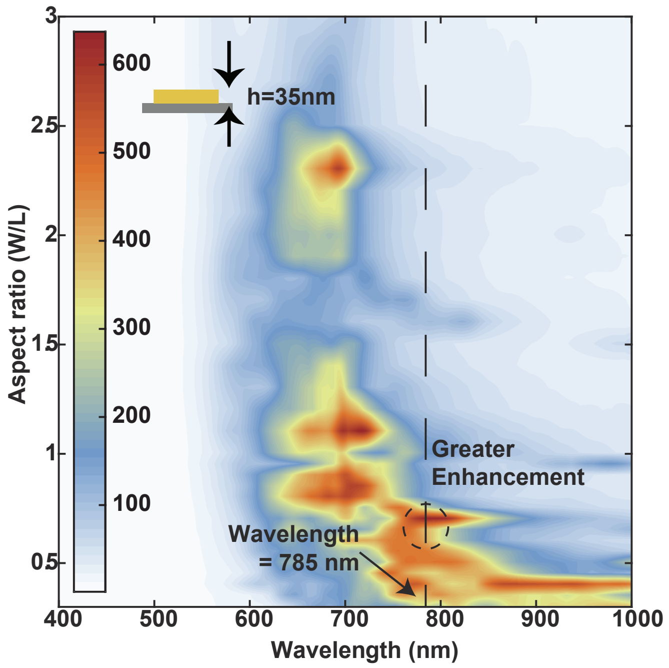

Fig. 3 Electrical field enhancement map for various aspect ratio when the gold dimer height, h = 35 nm.

(as depicted in Fig. 2(iii) of the manuscript). Ga+ ion beam can also be used for milling, but we propose He+-FIB since it offers better capability for direct patterning of nanostructures in the sub-10 nm scale7.

(e) Stripping of the remaining Au layer: An adhesive layer such as Scotch tape with suitable strength can now be attached to the Au film and be used to strip away the remaining Au layer, that are outside the closed contour of the cardiac shaped structures. As discussed by Chen et. al.[1,3] in the mechanism of the SPL, due to the closed contour, the metallic layer inside it, is adhered to the substrate more strongly than the outside layer and thus the outside layer can be easily "peeled-off", creating an array of isolated nanoparticles.

Supplementary Text 3: Plasmonic Response of Heart-shaped Dimer

We studied the plasmonic response of the heart shaped dimer, for various aspect ratios and at different heights, to deduce the best parameter for the dimer shape. The peak wavelength shows a non-linear blue shift with increasing aspect ratio while the height has negligible effects(Fig.2-a). For an aspect ratio of 0.5-0.8, the peak wavelength is in the vicinity of 785 nm. The scattering-to-absorption ratio at different wavelength for various aspect ratios is given in Fig.2-b.

Supplementary Text 4: Dependence of electric field enhancement on the height of Nanoparticles

If the nanoparticle's height is decreased to 35 nm, the dependence of electric field enhancement on aspect ratio remains quite the same and heart-shaped nanoparticles with an aspect ratio of 0.75

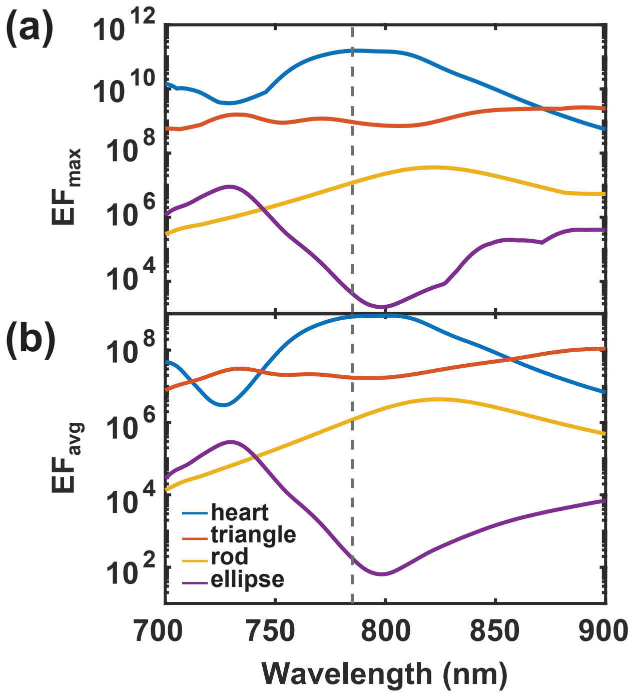

Fig. 4 Comparison of SERS Enhancement Factor for different shapes of structures.

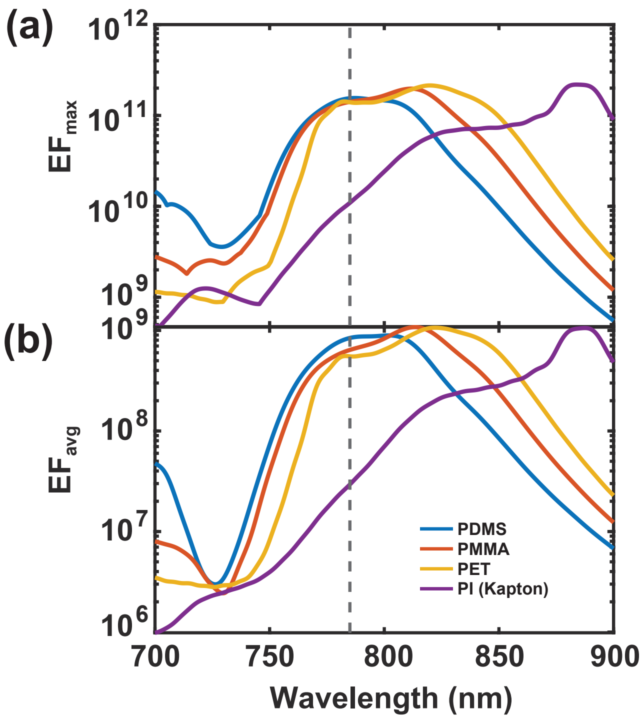

Fig. 5 Electrical field enhancement map for various aspect ratio when the gold dimer height, h = 35 nm.

or less produce high field enhancement close to the excitation wavelength (785 nm) analogous to the nanoparticle with a 45 nm height. The electric field is magnified more than 600 times at a ratio of roughly 0.7. Reducing the height causes a very small amount of change. More significantly, even though the level of enhancement is about the same, lowering the height of the NPs may result in a smaller hotspot. Table. S1 shows the derived parameters for different aspect ratios. As seen from the table, for a decreasing value of a, b and c the aspect ratio increases.

Supplementary Text 5: Comparison of SERS performance with different shapes

In our study, we conduct an extensive analysis of the SERS performance by evaluating various shapes of nanoparticle dimers, including rod-like, triangular, elliptical, and our proposed heart-shaped nanoparticles. The results, as shown in Fig. S4, clearly demonstrate that the heart-shaped nanoparticles exhibit orders of magnitude higher SERS enhancement compared to the other highly sensitive structures. Both the maximum enhancement factor (EF_{max}) and the average enhancement factor (EF_{avg}) are calculated over a volume of 10 nm $\times$ 10 nm $\times$ 10 nm. These findings further support the superior performance of our proposed heart-shaped nanoparticles in achieving enhanced SERS signals.

Supplementary Text 6: Comparison of SERS performance on different substrates

In addition to PDMS, we have evaluated the SERS performance of our proposed heart-structured nanoparticles on three other commonly used substrates in wearable sensing applications. Polyethylene terephthalate (PET) is a widely employed polymeric material in the healthcare industry due to its biocompatibility, high homogeneity, and mechanical strength[4]. Zang et al.[5] recently demonstrated the use of an Au nanoworm-covered PET substrate for SERS applications, achieving an enhancement factor on the order of 10^8. Polymethyl methacrylate (PMMA) is another flexible substrate that has been utilized in SERS applications. Gushiken et al.[6] demonstrated a SERS substrate comprising Au nanoparticles and PMMA. Additionally, polyimides (PI), commercially available as Kapton, are widely used due to their flexibility and biocompatibility.

To assess the SERS enhancement on these four substrates (PDMS, PMMA, PET, and PI), we calculated the enhancement factors for our proposed heart-shaped nanoparticles. The comparison of SERS enhancement factors is presented in Fig. S5. The refractive indices for PMMA and PET were obtained from Zhang et al.[2], and for PI (Kapton), they were referenced from French et al.[7].

Notes and references

1 Y. Chen, Q. Xiang, Z. Li, Y. Wang, Y. Meng and H. Duan, *Nano Letters*, 2016, **16**, 3253–3259.

2 X. Zhang, J. Qiu, X. Li, J. Zhao and L. Liu, *Appl. Opt.*, 2020, **59**, 2337–2344.

3 Y. Chen, Q. Xiang, Z. Li, Y. Wang, Y. Meng and H. Duan, *Nano Lett.*, 2016, **16**, 3253–3259.

4 M. Xu, D. Obodo and V. K. Yadavalli, *Biosensors and Bioelectronics*, 2019, **124-125**, 96–114.

5 S. Zang, H. Liu, Q. Wang, J. Yang, Z. Pang, K. Liu, S. Cai and

Table S1 | Parameter a, b and c associated with aspect ratios, AR = 0.3 to AR = 2.0, of the heart shaped nanoparticle

AR	a	b	c
0.3	99.1071	0.0662	102.3028
0.4	86.8220	0.0574	87.3940
0.5	77.0798	0.0513	78.8656
0.6	69.9288	0.0468	72.5213
0.7	65.7702	0.0434	65.8953
0.8	61.5657	0.0406	61.5870
0.9	56.9235	0.0382	59.4232
1.0	54.8688	0.0363	55.3241
1.2	49.5535	0.0331	51.1514
1.4	45.2356	0.0306	48.1351
1.6	43.4110	0.0287	43.6970
1.8	40.0859	0.0270	42.2186
2.0	37.8210	0.0256	40.3040

X. Ren, *Materials Letters*, 2021, **294**, 129643.

6 N. K. Gushiken, G. T. Paganoto, M. L. Temperini, F. S. Teixeira and M. C. Salvadori, *ACS Omega*, 2020, **5**, 10366–10373.

7 R. H. French, J. M. Rodriguez-Parada, M. K. Yang, R. A. Derryberry, M. F. Lemon, M. J. Brown, C. R. Haeger, S. L. Samuels, E. C. Romano and R. E. Richardson, 2009 34th IEEE Photovoltaic Specialists Conference (PVSC), 2009, pp. 000394–000399.
